# Online adaptive radiation therapy for muscle-invasive bladder cancer: short-course and high-precision definitive treatment for elderly or medically fragile patients

**DOI:** 10.1007/s11604-025-01885-4

**Published:** 2025-10-10

**Authors:** Peter J. K. Tokuda, Rihito Aizawa, Hiraku Iramina, Takahsi Ogata, Hideaki Hirashima, Yuki Kita, Takayuki Sumiyoshi, Takashi Kobayashi, Takashi Mizowaki

**Affiliations:** 1 Department of Radiation Oncology and Image-Applied Therapy, Graduate School of Medicine, Kyoto University, 54 Shogoin-Kawahara-Cho, Sakyo-Ku, Kyoto, Kyoto 606-8507 Japan; 2 Department of Urology, Graduate School of Medicine, Kyoto University, Kyoto, Japan

**Keywords:** Cone-beam computed tomography, Dementia, Elderly patients, Muscle-invasive bladder cancer, Online adaptive radiation therapy

## Abstract

**Purpose:**

External-beam radiation therapy is a treatment option for muscle-invasive bladder cancer (MIBC), which enables bladder preservation and is applicable to elderly or medically fragile patients. Since the accuracy of conventional intensity-modulated radiation therapy is vulnerable to even small daily changes, online adaptive radiation therapy (ART) may be a more appropriate option. This study aimed to evaluate the feasibility of cone-beam computed tomography (CBCT)-based online ART for MIBC in elderly or medically fragile patients.

**Materials and methods:**

We applied CBCT-based online ART to treat two elderly patients with dementia and N0 M0 MIBC, who were deemed poor candidates for surgery or systemic therapies. A cumulative dose of 55 Gy in 20 fractions over 4 weeks was prescribed. Online ART was administered using Ethos™ Therapy.

**Results:**

Both patients were able to follow the prescribed protocols with assistance (e.g., bladder voiding or filling and maintaining the treatment position), although full adherence was hindered by dementia. Nonetheless, they successfully completed the entire treatment regimen with manageable acute toxicities. The median treatment session duration was 23 (range 14–50, interquartile range 19–30) min. The median volume receiving at least 90% of the prescribed dose (V_90%_) of the bowels was 1.25 (range 0.01–6.77) cm^3^ in the adapted plans and 24.78 (range 6.80–90.72) cm^3^ in the scheduled plan (*P* < 0.001) in the first patient, and 0.13 (range 0.00–2.96) cm^3^ in the adapted plans and 9.60 (range 4.65–18.62) cm^3^ in the scheduled plan (*P* < 0.001) in the second patient, respectively. A significant volume of the bowels was spared from receiving high doses in the adapted plans without compromising the target dose coverage.

**Conclusions:**

CBCT-based online ART was considered a feasible therapeutic option for elderly or medically fragile patients with MIBC.

## Introduction

Muscle-invasive bladder cancer (MIBC) is a refractory malignancy. For its treatment, external-beam radiation therapy (EBRT) is a therapeutic option that acts as both bladder-preserving treatment [1–6] and an alternative to radical cystectomy in some patients due to an advanced age or a poor general condition [7]. Although intensity-modulated radiation therapy (IMRT) facilitates safe dose delivery by modifying the dose distribution, the accuracy of conventional IMRT is vulnerable to even small changes in the setup or treatment position and target shape, especially given patients' potentially limited capacity to follow pre-treatment protocols and the bladder's variable filling state. Considering the significant inter-fractional alteration in the size, shape, or location of the bladder [8], it is often difficult to apply conventional IMRT for MIBC.

Online adaptive radiation therapy (ART) is one of the key technical innovations in image-guidance methods for EBRT; it facilitates tailoring of the treatment plan to more appropriately complement changes in anatomical geography by re-creating the treatment plan prior to each session based on radiographic images [9–11]. This article focuses on our technical scheme and experiences of cone-beam computed tomography (CBCT)-based online ART for two elderly patients with dementia and N0 M0 MIBC, who were deemed poor candidates for surgery or systemic therapies.

## Materials and methods

### Patients

Two patients (81-year-old male with T3bN0M0 MIBC; 80-year-old female with T3a–bN0M0 MIBC) were referred to our department to receive EBRT for locally advanced MIBC. They underwent transurethral resection of bladder tumor, which resulted in incomplete resection of the tumor. They were deemed poor candidates for radical cystectomy or chemotherapy due to their poor general conditions and concomitant illnesses.

### Radiation therapy

Moderately hypofractionated IMRT using online ART was administered, as conventional long-course EBRT was deemed impractical due to the patients' dementia. A cumulative dose of 55 Gy in 20 fractions over 4 weeks was prescribed, in which the whole bladder was irradiated in the initial 16 fractions, followed by boost irradiation of the tumor involving 4 fractions.

**Fig. 1:**
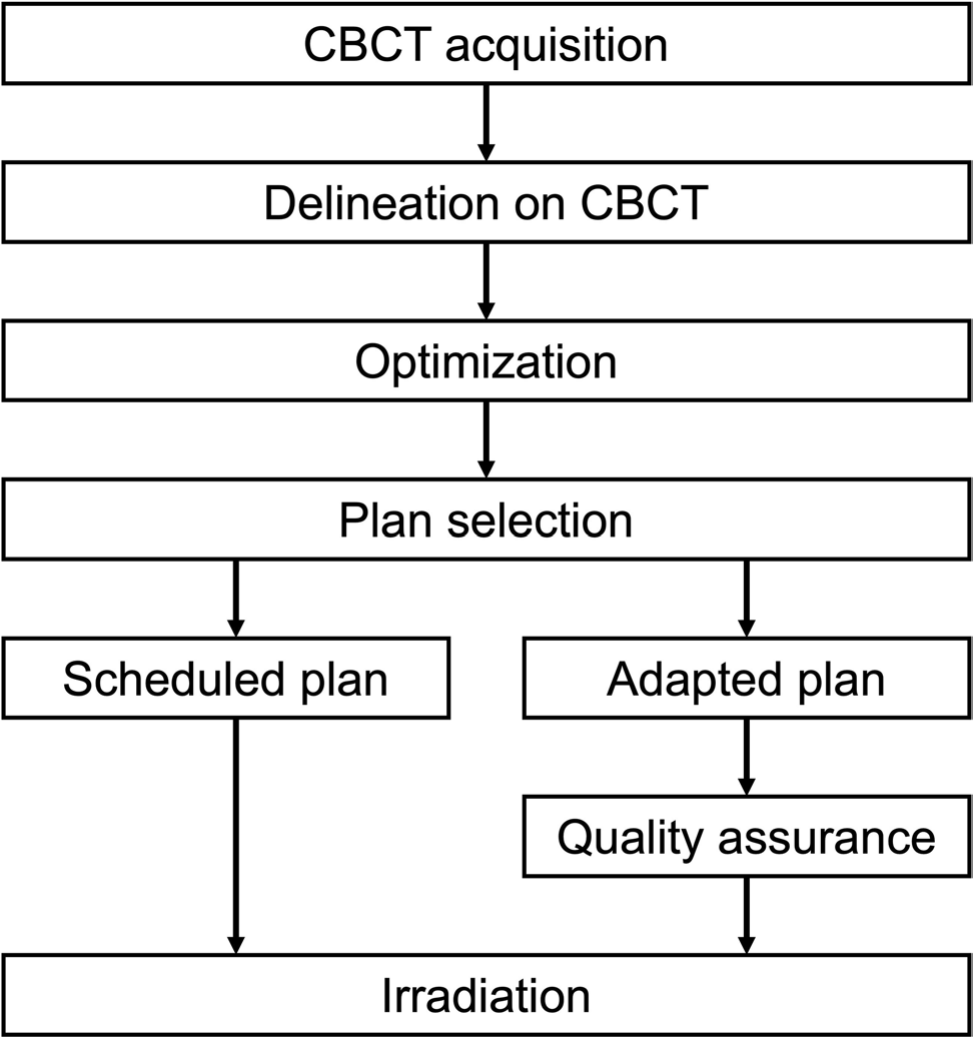
Standardized workflow of online ART

Online ART was administered using Ethos™ Therapy (Varian Medical Systems, Palo Alto, CA, USA), a CBCT-based radiation therapy treatment system that incorporates artificial intelligence and machine-learning. The schema of our workflow is shown in Fig. 1. In this study, “adapted plans” refer to daily re-optimized IMRT plans prepared using online ART, while the “scheduled plan” refers to the non-adapted IMRT plan prepared in advance as a backup in case adaptation was not feasible. An original scheduled plan was generated from a conventional simulation computed tomography (CT). The gross tumor volume (GTV) was defined as the remaining tumor. For the initial 16 fractions (initial plans), the clinical target volume (CTV) included the entire bladder, and the planning target volume (PTV) was generated by adding an 8-mm isotropic margin to the CTV. During the latter 4 fractions (boost plans), the CTV was defined as the tumor (i.e., the GTV), and the PTV was created by applying a 5–8-mm margin to the CTV. Post-void irradiation was recommended for the initial 16 fractions, and full-bladder irradiation for the subsequent 4 fractions; however, adherence to these instructions was considered challenging for the two patients due to cognitive impairment associated with dementia.

All treatments employed 6-MV flattening filter-free photon beams delivered via a seven-field fixed-gantry IMRT configuration. The prescription dose was defined as PTV D50. In regions where the bowels overlapped with the PTV, the dose was reduced by approximately 5–10%.

Prior to each treatment fraction, a CBCT acquisition with image quality sufficient for treatment planning was obtained, and target volumes and organs at risk were delineated on the CBCT. Ethos™ Therapy generated an adapted treatment plan for each session by re-optimizing the original plan, which was initially created using simulation CT, with the same clinical objectives. This adaptation was based on the CBCT acquired at the beginning of each treatment session. In addition, the scheduled plan was recalculated on the daily CBCT for reference, with the simulation CT automatically aligned to the day's CBCT based on the PTV. The system allowed selection between the adapted and scheduled plans. An automated quality assurance procedure was performed prior to irradiation when the adapted plan was chosen.

### Statistical analysis

We evaluated acute adverse events according to Common Terminology Criteria for Adverse Events v5.0.

To evaluate the treatment plans, we conducted pairwise comparisons using the Wilcoxon signed-rank test for several dosimetric parameters, including D_98%_ (the dose received by at least 98%) and D_95%_ for the CTV and PTV, as well as V_100%_ (the volume receiving at least 100% of the prescribed dose), V_95%_, V_90%_ and V_50%_ for the bowels (small intestine and colon) and rectum. These comparisons were performed for the adapted and scheduled plans across the initial 16 fractions in two patients separately. A *P* value of less than 0.025, following the Bonferroni correction for multiple comparisons (0.05/2), was considered statistically significant. All statistical analyses were performed using EZR version 1.61 [12], a graphical user interface for R version 4.1.2 (R Foundation for Statistical Computing, Vienna, Austria).

## Results

### Radiation therapy

Adapted plans were utilized in all treatment sessions. Instruction and guidance for EBRT were reiterated at each treatment session, and both patients were able to follow the prescribed protocols with assistance (e.g., bladder voiding or filling and maintaining the treatment position), although full adherence was hindered by dementia. Nonetheless, they successfully completed the entire treatment regimen.

For the first patient, CBCT was performed at the beginning of each treatment session, immediately before irradiation (after creation of the adapted plan), and at the end of the session, and changes in bladder shape and position were confirmed to remain within the PTV margins. For the second patient, in light of the experience with the first patient, CBCT was performed solely at the beginning of each treatment session, considering the patient's restlessness.

Both patients completed the entire planned treatment course with manageable acute toxicities: only grade 1 cystitis noninfective was observed in one patient, but no toxicities of grade 2 or higher were noted. The median treatment session duration was 23 (range 14–50, interquartile range 19–30) min.

### Dosimetric comparison between the adapted and scheduled plans

**Fig. 2:**
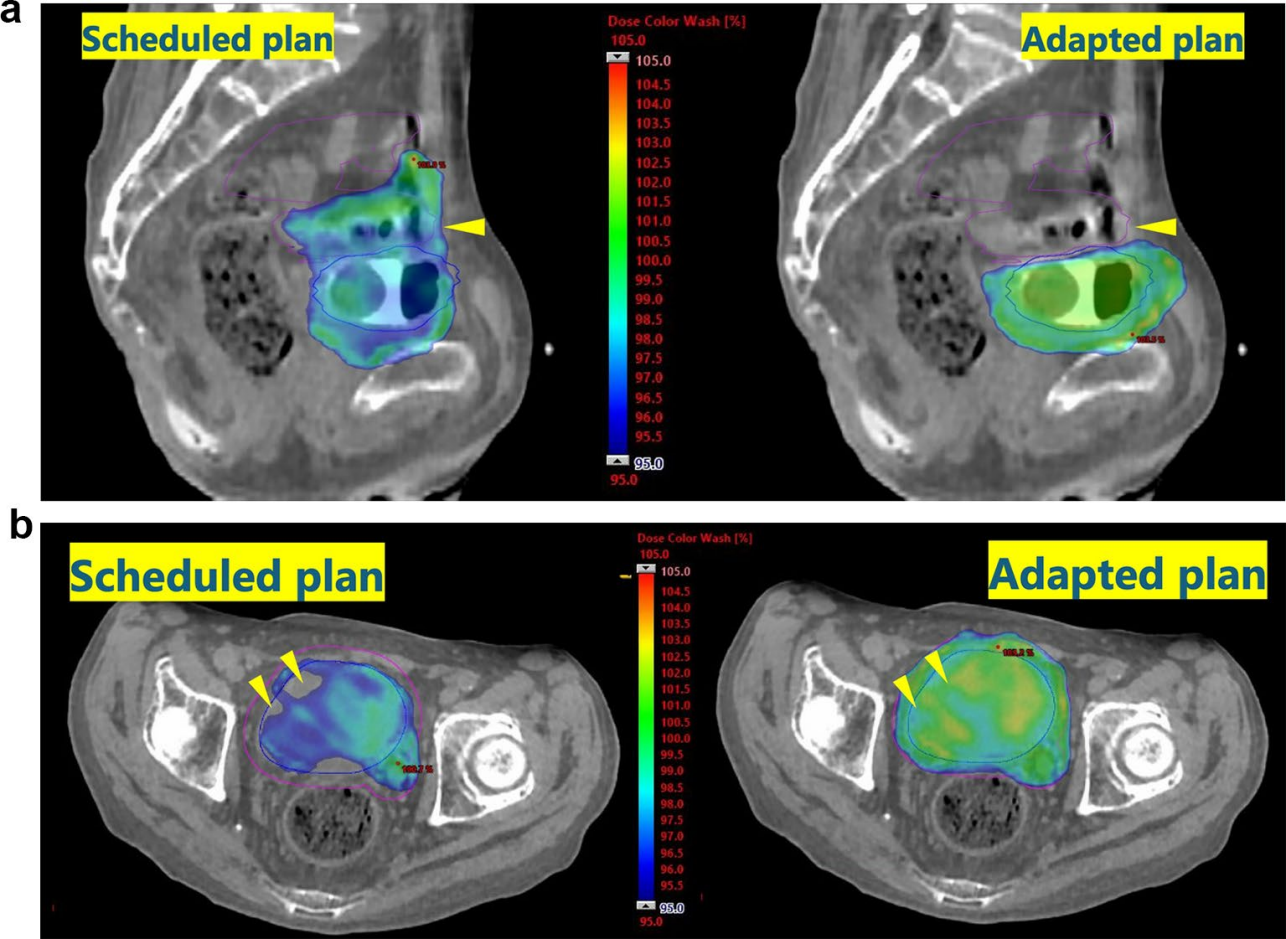
Dose distributions of the scheduled plan (left) and adapted plans (right) from typical sessions are shown. Colored areas received more than 95% (2.61 Gy per fraction) of the prescribed dose. The adapted plans showed greater conformity with the bladder and facilitated more deliberate avoidance of the bowels. In some sessions, the bladder was smaller than during computed tomography simulation, risking unnecessary irradiation of the bowels if the scheduled plan had been applied. A sagittal slice of the scheduled plan (left) versus an adapted plan (right) showed a marked difference in radiation doses to the bowels (purple) (**A**). In other sessions, the bladder was larger, potentially leading to insufficient radiation coverage of the target, although there was no significant difference when analyzed across all 16 initial sessions. An axial slice of the scheduled plan (left) versus another adapted plan (right) showed a difference in radiation doses to the bladder (blue) (**B**)

A significant volume of the bowels was spared from receiving high doses in the adapted plans without compromising the dose coverage of the PTV (Fig. 2). The details of the dose–volume histogram are shown in Table 1. For instance, in the initial 16 fractions, the median V_90%_ of the bowels was 1.25 (range 0.01–6.77) cm^3^ in the adapted plans and 24.78 (range 6.80–90.72) cm^3^ in the scheduled plan (*P* < 0.001) in the first patient, and 0.13 (range 0.00–2.96) cm^3^ in the adapted plans and 9.60 (range 4.65–18.62) cm^3^ in the scheduled plan (*P* < 0.001) in the second patient, respectively; the *P* values for D_95%_ of the PTV were 0.252 for the first patient and 1.000 for the second patient.

**Table 1:** Dosimetric comparison between adaptive plans and scheduled plans

			Patient 1			Patient 2		
			Adapted plans	Scheduled plan	*P* value	Adapted plans	Scheduled plan	*P* value
CTV	Volume	cm^3^	93.95 (57.82–195.50)			66.32 (52.84–74.44)		
	D_98%_	%	96.88 (94.78–98.03)	95.56 (92.21–97.21)	< 0.001	89.77 (88.83–92.94)	89.89 (88.14–92.62)	0.623
	D_95%_	%	98.40 (97.82–99.01)	96.12 (93.05–97.81)	< 0.001	91.51 (89.40–96.02)	90.97 (88.80–95.28)	0.376
PTV	Volume	cm^3^	221.19 (161.86–379.14)			135.104 (110.971–149.794)		
	D_98%_	%	87.69 (87.16–88.46)	88.89 (75.46–95.67)	0.528	87.33 (86.56–87.92)	85.88 (75.55–89.19)	0.013
	D_95%_	%	90.30 (87.98–92.63)	91.66 (83.21–96.69)	0.252	87.80 (87.04–88.74)	88.31 (83.20–89.79)	1.000
Rectum	V_100%_	cm^3^	0.00 (0.00–0.00)	0.00 (0.00–2.49)	0.022	0.00 (0.00–0.00)	0.00 (0.00–0.00)	N/A
	V_95%_	cm^3^	0.13 (0.00–1.78)	1.74 (0.00–19.22)	0.015	0.00 (0.00–0.00)	0.00 (0.00–0.35)	0.059
	V_90%_	cm^3^	6.12 (0.13–16.03)	8.74 (0.06–35.01)	0.669	0.00 (0.00–0.265)	0.00 (0.00–1.40)	0.014
	V_50%_	cm^3^	93.42 (38.36–161.93)	98.25 (45.41–161.16)	0.144	24.20 (12.18–42.45)	26.10 (16.99–46.56)	0.211
Bowels	V_100%_	cm^3^	0.00 (0.00–0.00)	4.10 (0.07–34.69)	< 0.001	0.00 (0.00–0.00)	0.72 (0.00–4.11)	0.003
	V_95%_	cm^3^	0.01 (0.00–1.07)	17.72 (3.24–76.90)	< 0.001	0.00 (0.00–0.00)	2.51 (0.00–8.60)	< 0.001
	V_90%_	cm^3^	1.25 (0.01–6.77)	24.78 (6.80–90.72)	< 0.001	0.13 (0.00–2.96)	9.60 (4.65–18.62)	< 0.001
	V_50%_	cm^3^	55.45 (18.97–99.56)	89.64 (39.80–161.08)	< 0.001	83.91 (54.11–103.00)	80.98 (66.29–101.07)	0.900

*CTV* clinical target volume, *PTV* planning target volume, *D*_*X%*_ the dose received by at least X%, *V*_*Y%*_ the volume receiving at least Y% of prescribed dose

## Discussion

We successfully applied moderately hypofractionated IMRT using CBCT-based online ART for elderly patients who were considered unsuitable candidates for conventional IMRT mainly due to their concomitant illnesses. To date, only a limited number of studies have been conducted on online ART for MIBC [13–15]; to our knowledge, no published study has exclusively focused on such patients. Unlike conventional EBRT, which adheres to a fixed plan throughout almost the entire treatment process, online ART updates or re-creates the treatment plan prior to each session based on newly acquired radiographic images (CBCT or magnetic resonance imaging [MRI]). Therefore, it can adjust the treatment plan by minimizing geographic uncertainty due to inter-fractional (day-to-day) patient-specific anatomical variations caused by changes in the shape of the target or surrounding normal tissues, potentially improving target volume coverage and minimizing doses to normal tissues. The phase 2 RAIDER trial investigated the feasibility of ART for T2–T4a MIBC, in which a total of 345 patients with MIBC were treated with standard-dose ART (SART; 55 Gy in 20 fractions or 64 Gy in 32 fractions), escalated-dose ART (DART; 60 Gy in 20 fractions or 70 Gy in 32 fractions to the tumor and 46 Gy in 20 fractions or 52 Gy in 32 fractions to the uninvolved bladder), or whole-bladder single-plan radiation therapy (WBRT; 55 Gy in 20 fractions or 64 Gy in 32 fractions) [16–18]. In that trial, although “online” ART was not utilized, the radiation therapy plans were selected daily from three plans (small, medium, and large) in the SART or DART groups. The 2-year locoregional disease control rates were 66% for WBRT or SART and 74.0% for DART. The rates of grade ≥ 3 radiation-induced late toxicities were: 4.3% for the 20-fraction SART group, 0% for the 32-fraction SART group, 1.7% for the 20-fraction DART group, and 0% for the 32-fraction DART group. As the merit of ART is maximized in cases showing marked day-to-day anatomical variations, online ART may represent a promising method for patients with MIBC, including elderly or medically fragile patients deemed unsuitable for conventional IMRT. Currently, the ARTIA-bladder multi-national prospective trial is on-going, in which daily online ART for MIBC is attempted in all patients [19]. Robust clinical data on the use of daily online ART for MIBC is expected to be generated in this trial.

One strength of CBCT-based online ART is its rapidness. Mangar SA et al. conducted an observational imaging study to investigate intra-fractional bladder volume changes using cine-MRI, and identified a linear increase in the bladder-filling rate over a 20-min observation period [20]. Specifically, the bladder volume increased by 1.6 cm^3^ per minute during treatment sessions. This suggests that intra-fractional bladder volume changes cannot be ignored, especially during extended treatment sessions. For our two patients, the complete online ART workflow was accomplished within a median duration of 23 min per treatment session. This was considered relatively efficient, given that the typical workflow duration for MRI-guided online ART has been reported to range from approximately 30–40 min [10]. Therefore, CBCT-based online ART, which has the potential to allow completion of online ART in a short time, would be beneficial as it minimizes any potential intra-fractional motion of the target.

Our patients were treated with moderately hypofractionated EBRT consisting of 55 Gy in 20 fractions over 4 weeks, as used in the hypofractionation arms of the phase 3 BC2001 trial and the phase 2 RAIDER trial [16, 21]. Although these trials prescribed 55 Gy in 20 fractions to the whole bladder, in the present study, the PTV was reduced during the boost phase in the latter 4 fractions to minimize the risk of quality-of-life deterioration due to bladder contraction. The higher treatment accuracy due to online ART enabled short-course and high-precision definitive treatment even for such patients. The application of ultra-hypofractionated EBRT has been attempted to reduce both patients' and clinicians' burden [17, 22]. As accurate dose delivery is indispensable for minimizing radiation-induced toxicities, online ART may also play an important role in stereotactic body radiation therapy. Hafeesz et al. performed a phase 2 study to assess the feasibility of ultra-hypofractionated radiation therapy for MIBC using offline ART, in which a dose of 36 Gy in 6 weekly fractions was employed to irradiate the whole bladder [17]. In that study, 55 patients with T2–4 Nx–2 M0–1 MIBC who were not suitable for cystectomy or daily radiation therapy were enrolled, and the population consisted of advanced-age patients (median of 86 years). As a result, 87% of the patients completed the treatment course, and radiation-induced toxicities were relatively mild. Specifically, regarding acute genitourinary toxicities, although rates of grades 2 and 3 toxicities were 40% and 18% during treatment, they decreased to 20% and 5% (of the assessed patients) at 6 weeks after the completion of radiation therapy, respectively. Regarding acute gastrointestinal toxicities, rates of grades 2 and 3 toxicities were 38% and 4%, respectively. Grades 2 and 3 late toxicities were observed in only 13% and 4.3% at 12 months after radiation therapy, respectively, and no grade 4 toxicities were noted at any time. The results suggest that ultra-hypofractionated radiation therapy using ART is feasible for this aged population. Thus, overall, online ART has the potential to alter the concept of definitive treatment for MIBC.

This study had several limitations, including its retrospective design and small sample size. CBCT data were unavailable for the second patient immediately prior to irradiation (after the adapted plan was created) and at the end of the treatment session, which restricts our ability to fully evaluate inter-fractional anatomical variations for this patient. In addition, further multi-institutional studies are needed to validate our results as this study was conducted at a single institution. Despite these limitations, our findings provide important insights into the feasibility and accuracy of online ART in this clinical context.

In conclusion, CBCT-based online ART was considered a feasible therapeutic option for elderly or medically fragile patients with MIBC. Given the comparatively aged MIBC population and growing demand for short-course EBRT, our technical scheme, experiences, and perspectives warrant sharing among urologists and radiation oncologists.

## Abbreviations

ART: Adaptive radiation therapy
CBCT: Cone-beam computed tomography
CT: Computed tomography
CTV: Clinical target volume
D_X%_: The dose received by at least X%
EBRT: External-beam radiation therapy
GTV: Gross tumor volume
IMRT: Intensity-modulated radiation therapy
MIBC: Muscle-invasive bladder cancer
MRI: Magnetic resonance imaging
PTV: Planning target volume
V_Y%_: The volume receiving at least Y% of prescribed dose
